# Supplementary material for: The causal role of circulating inflammatory markers in osteoporosis: a bidirectional Mendelian randomized study
Source: Front Immunol. 2024 Jul 18;15:1412298. doi: 10.3389/fimmu.2024.1412298 (PMC11291241; doi:10.3389/fimmu.2024.1412298)
Supplement: Supplementary file 1 [file DataSheet_1.zip › supplementary material/OSTEOPOROSIS-CIM/GCST90274845.tsv.gz_leave_one_out.pdf]

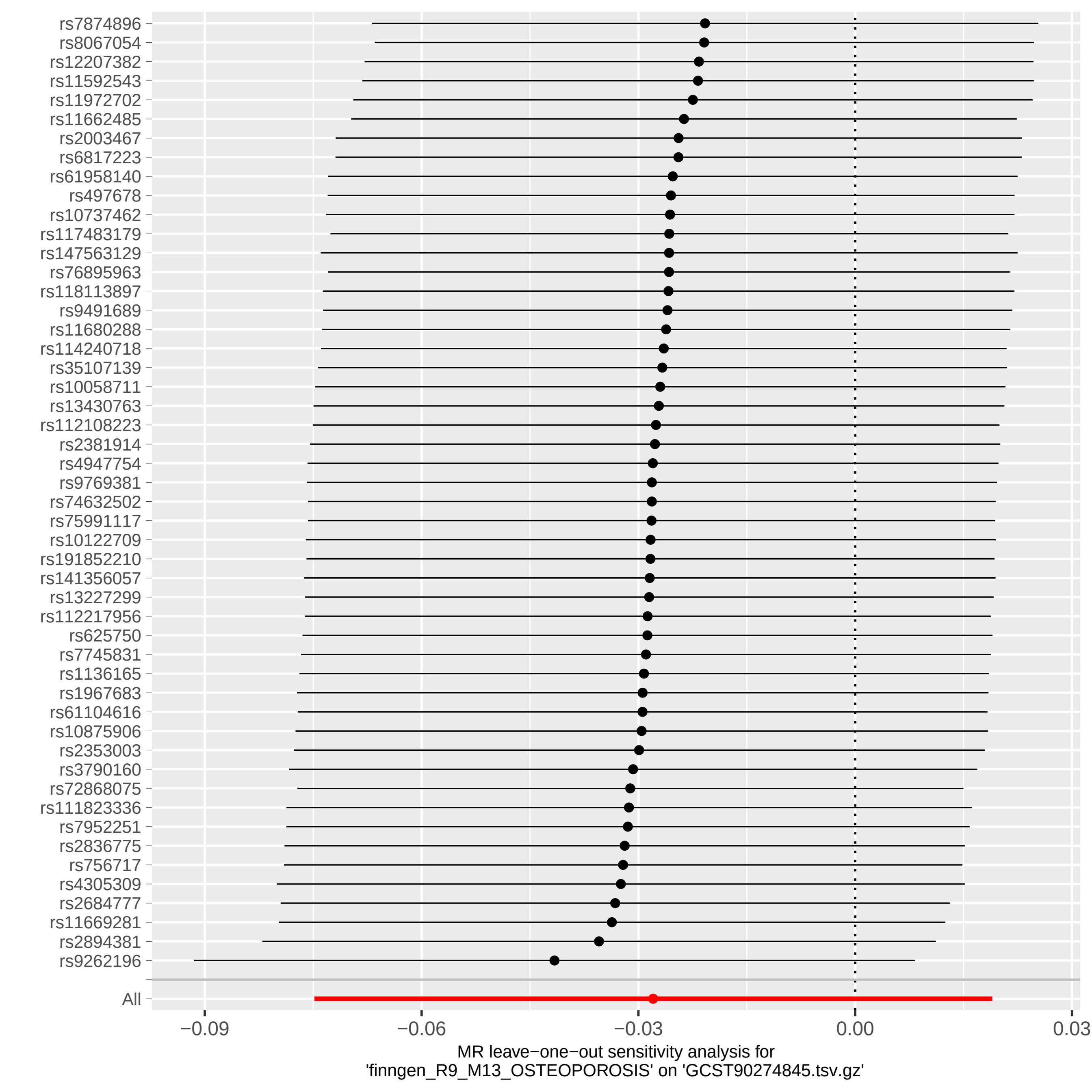

rs7874896
rs8067054
rs12207382
rs11592543
rs11972702
rs11662485
rs2003467
rs6817223
rs61958140
rs497678
rs10737462
rs117483179
rs147563129
rs76895963
rs118113897
rs9491689
rs11680288
rs114240718
rs35107139
rs10058711
rs13430763
rs112108223
rs2381914
rs4947754
rs9769381
rs74632502
rs75991117
rs10122709
rs191852210
rs141356057
rs13227299
rs112217956
rs625750
rs7745831
rs1136165
rs1967683
rs61104616
rs10875906
rs2353003
rs3790160
rs72868075
rs111823336
rs7952251
rs2836775
rs756717
rs4305309
rs2684777
rs11669281
rs2894381
rs9262196
All
−0.09
−0.06
−0.03
0.00
0.03
MR leave−one−out sensitivity analysis for
'finngen_R9_M13_OSTEOPOROSIS' on 'GCST90274845.tsv.gz'
